# Sample checklist of Gastropoda and Bivalvia in Cham Islands, Vietnam

**DOI:** 10.3897/BDJ.7.e32930

**Published:** 2019-02-19

**Authors:** Do Van Tu, Le Hung Anh, Luu The Anh, Takenori Sasaki, Tran Anh Tuan

**Affiliations:** 1 Graduate University of Science and Technology, Vietnam Academy of Science and Technology, 18 Hoang Quoc Viet, Cau Giay, Hanoi, Vietnam Graduate University of Science and Technology, Vietnam Academy of Science and Technology 18 Hoang Quoc Viet, Cau Giay, Hanoi Vietnam; 2 Institute of Ecology and Biological Resources, Vietnam Academy of Science and Technology, 18 Hoang Quoc Viet, Cau Giay, Hanoi, Vietnam Institute of Ecology and Biological Resources, Vietnam Academy of Science and Technology 18 Hoang Quoc Viet, Cau Giay, Hanoi Vietnam; 3 Institute of Geography, Vietnam Academy of Science and Technology, 18 Hoang Quoc Viet, Cau Giay, Hanoi, Vietnam Institute of Geography, Vietnam Academy of Science and Technology 18 Hoang Quoc Viet, Cau Giay, Hanoi Vietnam; 4 University Museum, The University of Tokyo, Tokyo, Japan University Museum, The University of Tokyo Tokyo Japan

**Keywords:** Sample checklist, Marine mollusc, Cham Islands, Vietnam

## Abstract

**Background:**

Cham Islands (Cu Lao Cham) is a group of 8 small islands in the Quang Nam province, Central Vietnam. There is only one study that mentioned the diversity of marine molluscs in this area. However, the data on species composition have not been digitised and not stored or shared for other purposes. Our paper aims to share the checklist of marine mollusc species (Bivalvia and Gastropoda) species that we collected from the littoral zone of Cham Islands in May 2017. This is the first digitised and online data of marine molluscs in Vietnam. It is very important for researchers in various fields such as the structure and function of ecology and biodiversity monitoring.

**New information:**

This study provides a checklist of the marine bivalves and snails in the Cham Islands of Vietnam. Moreover, this first widely shared data of biodiversity in Vietnam can trigger biodiversity data papers in this data-poor country.

The data of this study will be important inputs for better understanding biodiversity on the Cham Islands and Vietnam as well as for forming the basis for monitoring, exploitation and conservation of biodiversity in this area.

In total, 145 taxa were recorded, 46 bivalve taxa and 99 snail taxa, from which 128 were identified to the species level and 17 were identified to the genus level. There are 116 new species records for the Cham Islands.

The specimens are currently deposited in the Institute of Ecology and Biological Resources (IEBR), Vietnam Academy of Science and Technology (VAST).

## Introduction

Cham Islands forms a part of Cu Lao Cham Marine Protected Area and has been recognised by UNESCO as a World Biosphere Reserve (Cu Lao Cham Marine Park) in 2009. There is only one study on macrobenthic fauna on the coral reefs that also mentioned marine molluscs in the Cu Lao Cham Sea (Tuyen 2013). However, its data on the species checklist was not digitised or stored in any database. Therefore, it was not widely used by other scientists. Our aim here is to show the list of snails (Gastropod) and bivalves (Bivalvia) that we found during our survey in May 2017 from Cham Islands. This checklist is the first formal biodiversity data of this group of islands of Vietnam.

## General description

### Purpose

This study aims:

1. to provide the list of snail and bivalve species distributed in Cham Islands;

2. to store and register the specimens for future studies.

The survey was conducted in May 2017 in the littoral zones of Cham Islands to the depths between 5 and 10 metres.

### Additional information

This is a database of the scientific names and the coordinates of collected specimens.

## Project description

### Title

Sample checklist of Gastropoda and Bivalvia in Cham Islands, Vietnam

### Personnel

The researchers involved in this project were Dr. Do Van Tu (sample collection, taxonomic identification, data management), Dr. Le Hung Anh (sample collection) and Dr. Tran Anh Tuan (map drawing) from the Institute of Ecology and Biological Resources and Dr. Luu The Anh (sample collection) from theInstitute of Geography. Both institutes are under the Vietnam Academy of Science and Technology. Moreover, Dr. Takenori Sasaki from the University Museum, The University of Tokyo, helped to verify the identification of all species.

### Study area description

Cham Archipelago consists of one large island surrounded by seven small islands covering a sea area of 15 square kilometres and it is located in Central Vietnam, 16 kilometres from the coast and 19 kilometres to the east of the ancient Hoi An town, Quang Nam province (source: https://en.wikipedia.org). The names of islands are the Hon Lao (Pearl), Hon Dai (Long), Hon Mo (Tomb), Hon Kho me (Big Dry), Hon Kho con (Small Dry), Hon La (Leaf), Hon Tai (Ear) and Hon Ong (East Wind). The coral reefs and seagrass beds are widely distributed in the shallow waters of Cu Lao Cham. The researchers have recorded 261 species of Scleractinian coral, 15 species of soft coral, 3 species of fire coral (Milleporidae), 1 species of blue coral (Helioporidae) and 2 species of horny coral (Antipatharia) in this area (Ngoc 2018).

**Figure 1.**
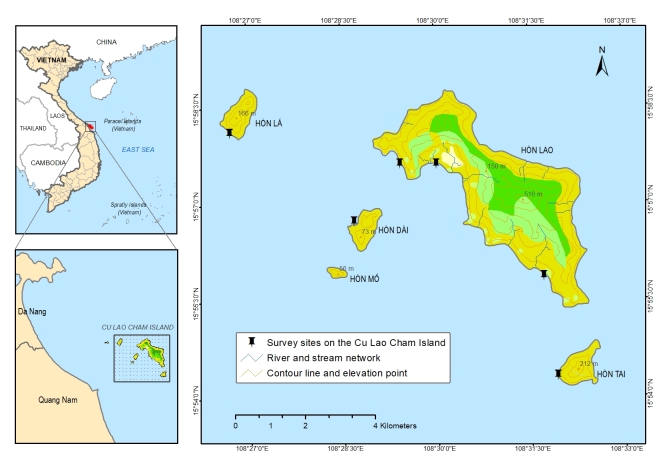
A map of the study area, the sampling sites are indicated.

We only surveyed around the large island (Hon Lao) and three other small islands (Hon Tai, Hon La, Hon Dai) (Fig. 1). The sampling sites were sandy beaches, rocky shores and coral reefs. The collection of specimens was approved by Management Board of Cu Lao Cham Marine Protected Area.

### Design description

This project is designed to obtain as many marine mollusc species as possible in the littoral zone of the Cham Islands.

### Funding

The project was funded by a grant from the Ministry of Science and Technology of Vietnam, Project code is ĐTĐL.XH-02/16.

## Sampling methods

### Study extent

The sampling sites were located in the littoral zone of Cham Islands (Fig. 1), including three sites (Bai Huong (Fig. 4), Bai Ong (Fig. 5), Bai Bac (Fig. 6)) at the large island (Hon Lao) and three sites at three other small islands (Hon Tai (Figs 7, 8), Hon La (Fig. 9), Hon Dai (Fig. 10).

### Sampling description

Marine molluscs (snails and bivalves) were collected by hand or scoop at the low tide in the different benthic habitats, including the sandy beaches, rocky shores and coral reefs by scuba divers. The sediments were sieved through a 1-mm mesh; the sieve residue was fixed in 95% Ethanol. In the laboratory, the samples were sorted and identified, where possible, to the species level based on taxonomic documents (Abbott and Dance 1986, Carpenter and Niem 1998, Okutani and (Ed.) 2017, Thach 2005, Thach 2007, Thach 2012, Thach 2016).

### Quality control

The scientific names in this paper are based on the identification by experienced experts in the field of the taxonomy of marine molluscs, namely, Dr. Takenori Sasaki from the University Museum, the University of Tokyo and Dr. Do Van Tu from Institute of Ecology and Biological Resources. All taxon names were checked against the Taxon Match tool in the World Register of Marine Species (WoRMS) in order to standardise and correct them, if necessary (http://www.marinespecies.org/aphia.php?p=match).

### Step description

NA

## Geographic coverage

### Description

The survey conducted in the Cham Islands including the large island (Hon La) and three other small islands (Hon Tai, Hon Dai, Hon La). Cham Islands belongs to Hoi An city, Quang Nam province. This island is located in Central Vietnam.

### Coordinates

15.896 and 15.984 Latitude; 108.437 and 108.55 Longitude.

## Taxonomic coverage

### Description

**Figure 2.**
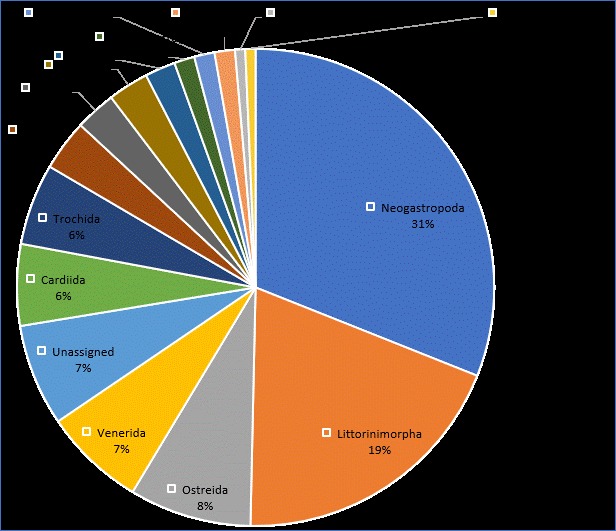
Taxonomic coverage (by taxonomic order).

**Figure 3.**
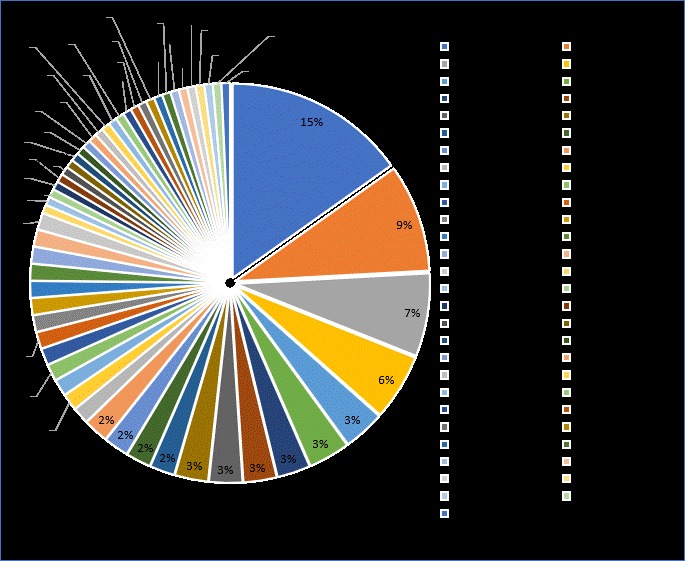
Taxonomic coverage (by taxonomic family).

We recorded 145 taxa of bivalves and snails (Table 1, Suppl. material 1). Of these, 116 species have been recorded for the first time in the Cu Lao Cham Island. Gastropoda is dominant with 35 families and 99 species while bivalves were represented by 20 families and 46 species. At the order level, the following were classified into Bivalvia: Arcida (4 species), Cardiida (8 species), Lucinida (3 species), Mytilida (5 species), Ostreida (12 species), Pectinida (4 species) and Venerida (10 species), Gastropoda: Caenogastropoda (2 species), Cycloneritida (2 species), Lepetellida (2 species), Littorinimorpha (28 species), Neogastropoda (45 species), Seguenziida (1 species), Siphonariida (1 species), Trochida (8 species) and unassigned (10 species) (Fig. 2.) The families with the highest species richness were Muricidae (22 species), Cypraeidae (13 species), Conidae (10 species) and Veneridae (8 species). The rest of the families were mostly represented by a single species (Fig. 3). Photos of 40 taxa were presented in Suppl. material 2.

## Traits coverage

### Data coverage of traits

PLEASE FILL IN TRAIT INFORMATION HERE

## Temporal coverage

### Notes

The field survey was conducted from 10-05-2017 through to 12-05-2017. The data were created from June 2017 until now. Some specimens could not be identified to the species level for the moment and the data will be updated in the future.

## Collection data

### Collection name

IEBR_Molluscs

### Collection identifier

Do Van Tu, Takenori Sasaki

### Parent collection identifier

IEBR

### Specimen preservation method

After being collected, the specimens were preserved in ethanol 95%.

## Usage rights

### Use license

Creative Commons Public Domain Waiver (CC-Zero)

### IP rights notes

This work is licensed under a Creative Commons Attribution (CC-BY) 4.0 License.

## Data resources

### Data package title

Checklist of marine mollusc in Cham Islands, Hoi An city, Quang Nam province, Vietnam

### Alternative identifiers

Do Van Tu

### Number of data sets

1

### Data set 1.

#### Data set name

Checklist marine mollusc in Cham Islands

#### Data format

Darwin Core

#### Number of columns

8

#### Description

The checklist is based on identifying the collected specimens to the species level where possible.

**Data set 1.** 

Column label	Column description
SCIENTIFIC_NAME	Complete scientific name including author and year
Specimen_number	The code of the sample is used for identifying the sample from which the record is derived. We also considered the specimen number as the voucher number.
Locality_Name	Name of locality where the sample was collected
Longtitude	The geographic longitude (in decimal degrees)
Latitude	The geographic latitude (in decimal degrees)
Collected_Date	DDMMYY
Family	The full scientific name of the family in which the taxon is classified
New_Record	New record for the Cham Islands

## Supplementary Material

Supplementary material 1 The list of Bivalvia and Gastropod recorded in Cham Island in May 2017 Data type: Occurrences
Brief description: The complete table of table 1
File: oo_255113.csv Do Van Tu, Takenori Sasaki, Le Hung Anh, Luu The Anh, Tran Anh Tuan

Supplementary material 2 Some photos of the species collected from Cham Islands, Vietnam, in May 2017 Data type: PFD file
Brief description: The file contains the photos of 40 species of Bivalvia and Gastropoda collected from Cham Islands
File: oo_261165.pdf Do Van Tu, Le Hung Anh, Luu The Anh, Takenori Sasaki, Tran Anh Tua

## Figures and Tables

**Figure 4.**
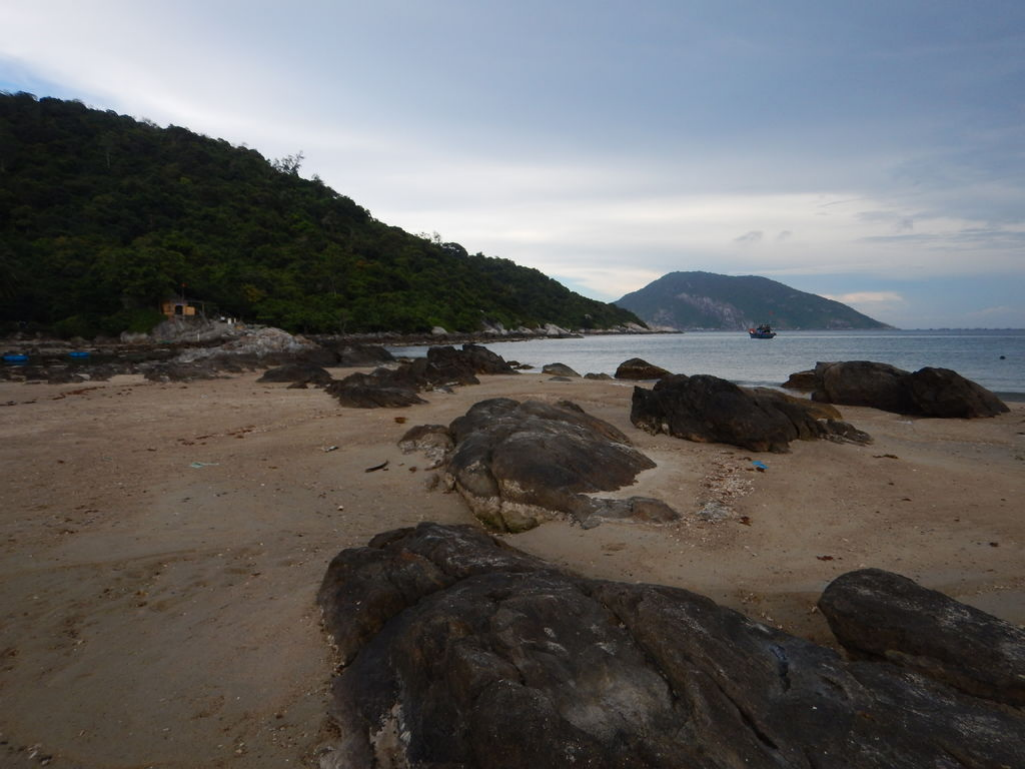
Bai Huong, Cu Lao Cham.

**Figure 5.**
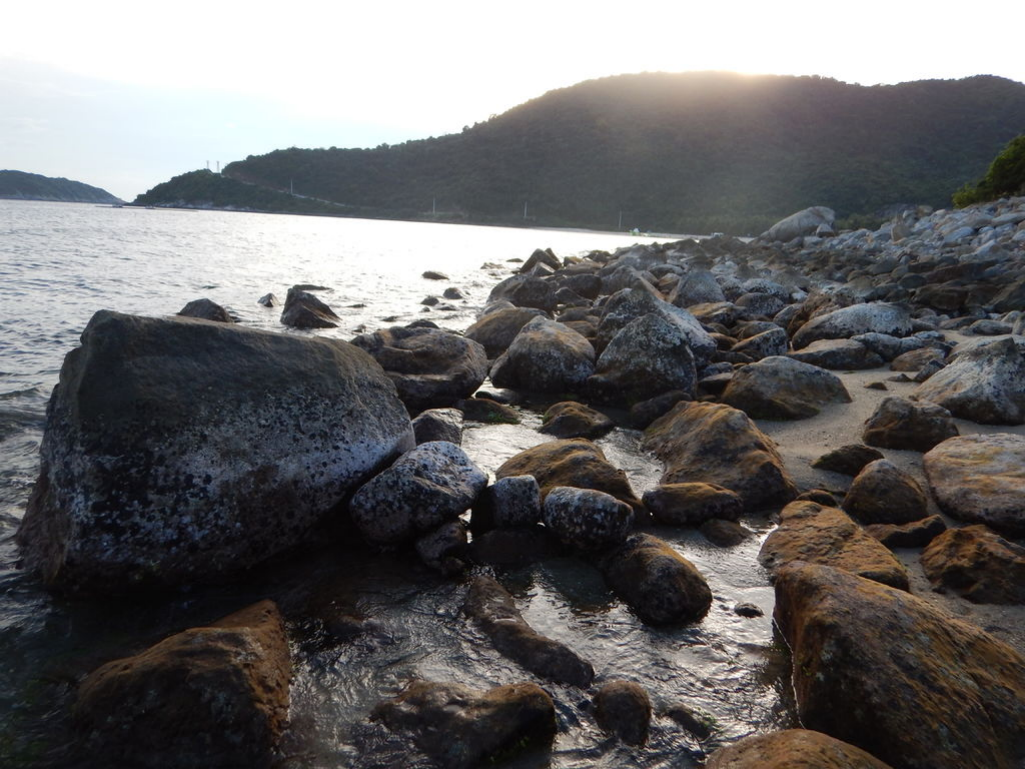
Bai Ong, Cu Lao Cham.

**Figure 6.**
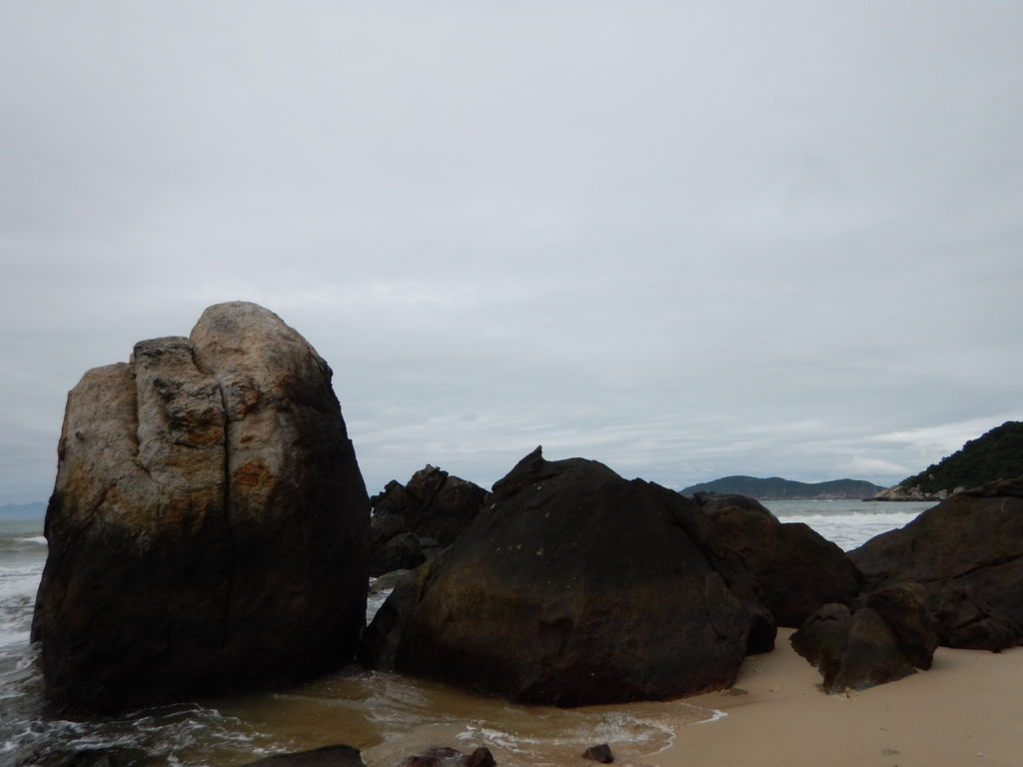
Bai Bac, Cu Lao Cham.

**Figure 7.**
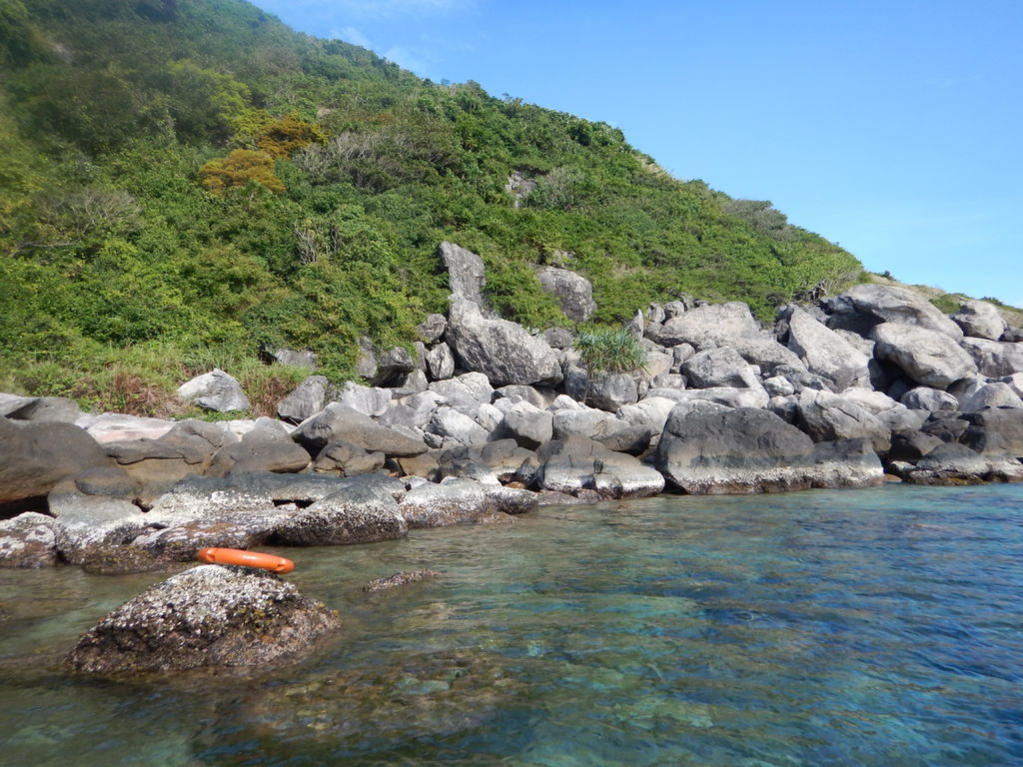
Hon Tai, Cu Lao Cham.

**Figure 8.**
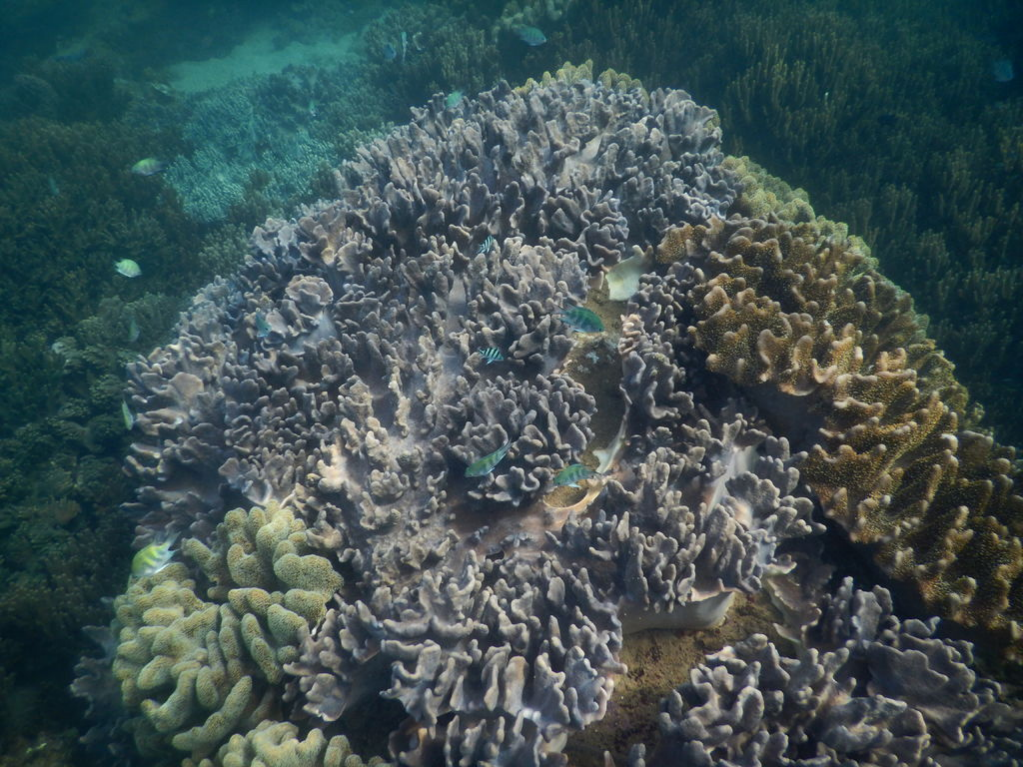
Coral reef in Hon Tai, Cu Lao Cham.

**Figure 9.**
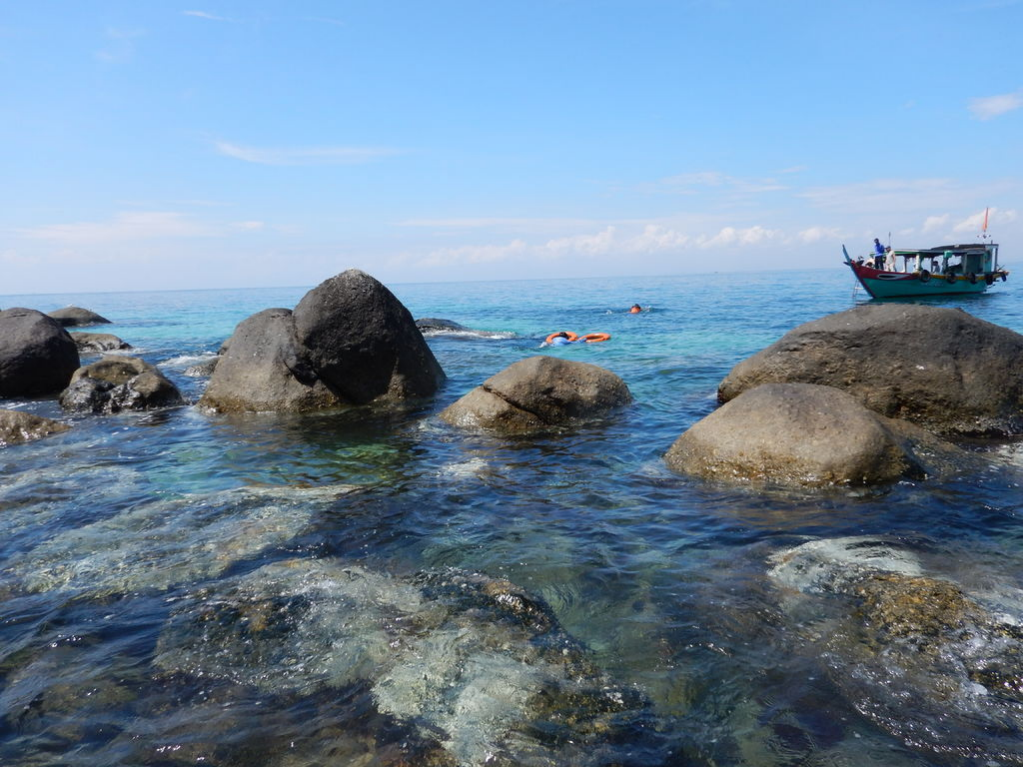
Hon La, Cu Lao Cham.

**Figure 10.**
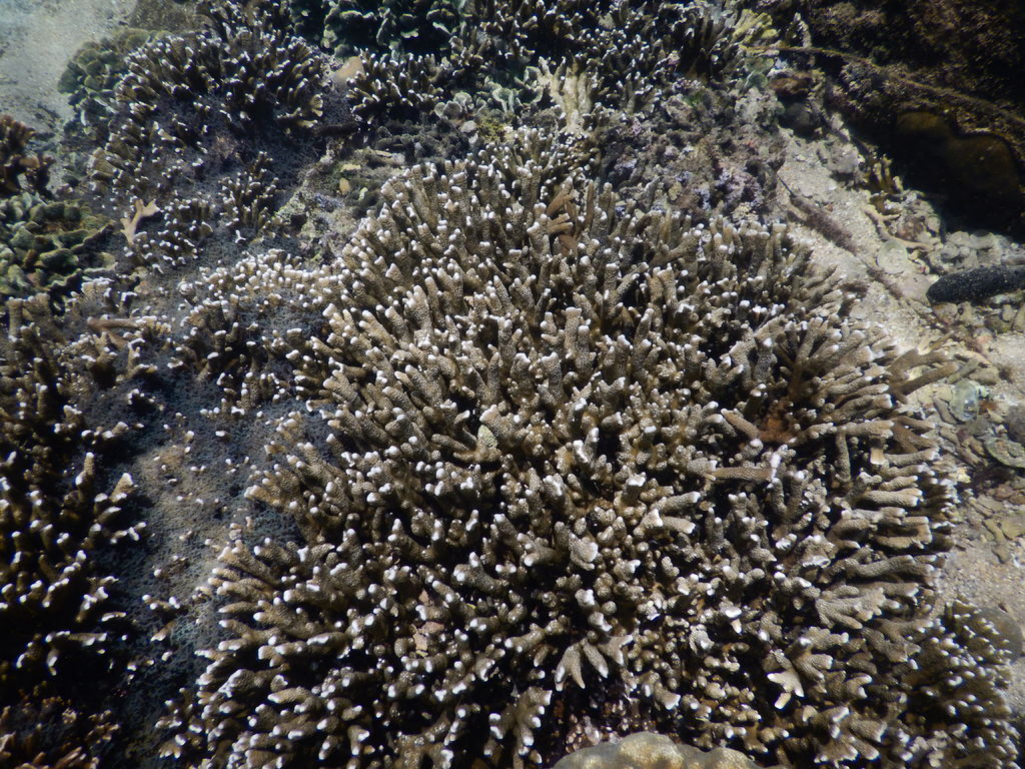
Coral reef in Hon Dai, Cu Lao Cham.

**Table 1.** The list of Bivalvia and Gastropod recorded in Cham Island in May 2017 (the species were presented in family order).

**SCIENTIFIC NAME**	**Family**	**Specimen number**	**Locality Name**	**Longtitude**	**Latitude**	**Date**	**New Record**
*Arca avellana* Lamarck, 1819	Arcidae	1	Bai Huong	108.52849	15.930946	2017/5/10	+
*Arca ventricosa* Lamarck, 1819	Arcidae	2	Bai Huong	108.52849	15.930946	2017/5/10	+
*Barbatia amygdalumtostum* (Röding, 1798)	Arcidae	3	Hon La	108.44516	15.96868	2017/5/10	+
*Barbatia foliata* (Forsskål in Niebuhr, 1775)	Arcidae	4	Hon La	108.44516	15.96868	2017/5/10	
*Tridacna crocea* Lamarck, 1819	Cardiidae	20	Hon Dai	108.47814	15.94558	2017/5/10	
*Vasticardium angulatum* (Lamarck, 1819)	Cardiidae	21	Bai Ong	108.50021	15.960193	2017/5/11	+
*Cardita variegata* Bruguière, 1792	Carditidae	19	Hon Tai	108.53193	15.90513	2017/5/10	
*Donax cuneatus* Linnaeus, 1758	Donacidae	18	Bai Huong	108.52849	15.930946	2017/5/10	+
*Asaphis violascens* (Forsskål in Niebuhr, 1775)	Psammobiidae	22	Bai Bac	108.49052	15.96038	2017/5/12	+
*Gari elongata* (Lamarck, 1818)	Psammobiidae	23	Bai Ong	108.50021	15.960193	2017/5/11	+
*Semele zebuensis* (Hanley, 1843)	Semelidae	25	Bai Ong	108.50021	15.960193	2017/5/11	+
*Macalia bruguieri* (Hanley, 1844)	Tellinidae	24	Bai Huong	108.52849	15.930946	2017/5/10	+
*Anodontia edentula* (Linnaeus, 1758)	Lucinidae	26	Bai Ong	108.50021	15.960193	2017/5/11	+
*Codakia tigerina* (Linnaeus, 1758)	Lucinidae	27	Bai Bac	108.49052	15.96038	2017/5/12	+
*Ctena bella* (Conrad, 1837)	Lucinidae	28	Bai Bac	108.49052	15.96038	2017/5/12	+
*Brachidontes* sp.	Mytilidae	5	Hon Tai	108.53193	15.90513	2017/5/10	+
*Lithophaga* sp.	Mytilidae	6	Hon Dai	108.47814	15.94558	2017/5/10	+
*Perna viridis* (Linnaeus, 1758)	Mytilidae	7	Bai Huong	108.52849	15.930946	2017/5/10	+
*Septifer bilocularis* (Linnaeus, 1758)	Mytilidae	8	Hon Dai	108.47814	15.94558	2017/5/10	
*Septifer* sp.	Mytilidae	9	Bai Bac	108.49052	15.96038	2017/5/12	+
*Hyotissa inermis* (G.B.Sowerby II, 1871)	Gryphaeidae	41	Bai Ong	108.50021	15.960193	2017/5/11	
*Hyotissa* sp.	Gryphaeidae	42	Bai Huong	108.52849	15.930946	2017/5/10	+
*Malleus albus* Lamarck, 1819	Malleidae	43	Bai Ong	108.50021	15.960193	2017/5/11	
*Crassostrea gigas* (Thunberg, 1793)	Ostreidae	37	Bai Huong	108.52849	15.930946	2017/5/10	+
*Dendostrea* sp.	Ostreidae	38	Bai Huong	108.52849	15.930946	2017/5/10	+
*Saccostrea glomerata* (Gould, 1850)	Ostreidae	39	Hon Tai	108.53193	15.90513	2017/5/10	+
*Saccostrea cf. mordax* (Gould, 1850)	Ostreidae	40	Bai Ong	108.50021	15.960193	2017/5/11	+
*Atrina* sp.	Pinnidae	36	Hon Tai	108.53193	15.90513	2017/5/10	+
*Amusium pleuronectes* (Linnaeus, 1758)	Pectinidae	29	Hon Tai	108.53193	15.90513	2017/5/10	+
*Mimachlamys sanguinea* (Linnaeus, 1758)	Pectinidae	30	Hon Dai	108.47814	15.94558	2017/5/10	+
*Plicatula australis* Lamarck, 1819	Plicatulidae	31	Bai Ong	108.50021	15.960193	2017/5/11	+
*Spondylus* sp.	Spondylidae	32	Hon Tai	108.53193	15.90513	2017/5/10	
*Isognomon* sp.	Pteriidae	33	Hon Tai	108.53193	15.90513	2017/5/10	+
*Pinctada martensii* (Dunker, 1872)	Pteriidae	34	Hon Dai	108.47814	15.94558	2017/5/10	
*Pinctada margaritifera* (Linnaeus, 1758	Pteriidae	143	Hon Dai	108.47814	15.94558	2017/5/10	
*Pteria penguin* (Röding, 1798)	Pteriidae	35	Hon Dai	108.47814	15.94558	2017/5/10	
*Chama limbula* Lamarck, 1819	Chamidae	45	Bai Bac	108.49052	15.96038	2017/5/12	+
*Atactodea striata* (Gmelin, 1791)	Mesodesmatidae	44	Bai Huong	108.52849	15.930946	2017/5/10	+
*Gafrarium dispar* (Holten, 1802)	Veneridae	10	Hon Tai	108.53193	15.90513	2017/5/10	+
*Gafrarium pectinatum* (Linnaeus, 1758)	Veneridae	11	Hon Tai	108.53193	15.90513	2017/5/10	+
*Globivenus toreuma* (Gould, 1850)	Veneridae	12	Bai Bac	108.49052	15.96038	2017/5/12	+
*Lioconcha fastigiata* (G. B. Sowerby II, 1851)	Veneridae	13	Bai Ong	108.50021	15.960193	2017/5/11	+
*Paphia undulata* (Born, 1778)	Veneridae	14	Bai Huong	108.52849	15.930946	2017/5/10	+
*Periglypta albocancellata* (M. Huber, 2010)	Veneridae	15	Hon Dai	108.47814	15.94558	2017/5/10	+
*Periglypta reticulata* (Linnaeus, 1758)	Veneridae	16	Hon Dai	108.47814	15.94558	2017/5/10	
*Venerupis aspera* (Quoy & Gaimard, 1835)	Veneridae	17	Bai Ong	108.50021	15.960193	2017/5/11	+
*Janthina umbilicata* d'Orbigny, 1841	Epitoniidae	46	Bai Huong	108.52849	15.930946	2017/5/10	+
*Planaxis sulcatus* (Born, 1778)	Planaxidae	47	Bai Ong	108.50021	15.960193	2017/5/11	+
*Nerita albicilla* Linnaeus, 1758	Neritidae	51	Bai Ong	108.50021	15.960193	2017/5/11	+
*Nerita balteata* Reeve, 1855	Neritidae	52	Bai Bac	108.49052	15.96038	2017/5/12	+
*Haliotis ovina* Gmelin, 1791	Haliotidae	54	Hon Tai	108.53193	15.90513	2017/5/10	
*Haliotis varia* Linnaeus, 1758	Haliotidae	55	Hon Tai	108.53193	15.90513	2017/5/10	
*Bufonaria rana* (Linnaeus, 1758)	Bursidae	98	Hon La	108.44516	15.96868	2017/5/10	+
*Bursa granularis* (Röding, 1798)	Bursidae	139	Bai Ong	108.50021	15.960193	2017/5/11	+
*Tutufa oyamai* Habe, 1973	Bursidae	145	Hon Dai	108.47814	15.94558	2017/5/10	+
*Lotoria lotoria* (Linnaeus, 1758)	Cymatiidae	142	Hon Dai	108.47814	15.94558	2017/5/10	+
*Bistolida ursellus* (Gmelin, 1791)	Cypraeidae	104	Hon Dai	108.47814	15.94558	2017/5/10	+
*Cypraea tigris* Linnaeus, 1758	Cypraeidae	105	Hon Tai	108.53193	15.90513	2017/5/10	+
*Erosaria erosa* (Linnaeus, 1758)	Cypraeidae	106	Hon La	108.44516	15.96868	2017/5/10	
*Erronea errones* (Linnaeus, 1758)	Cypraeidae	107	Hon Dai	108.47814	15.94558	2017/5/10	+
*Luria isabella* (Linnaeus, 1758)	Cypraeidae	110	Bai Huong	108.52849	15.930946	2017/5/10	+
*Lyncina carneola* (Linnaeus, 1758)	Cypraeidae	108	Hon Dai	108.47814	15.94558	2017/5/10	+
*Lyncina lynx* (Linnaeus, 1758)	Cypraeidae	109	Bai Ong	108.50021	15.960193	2017/5/11	+
*Mauritia arabica* (Linnaeus, 1758)	Cypraeidae	111	Hon La	108.44516	15.96868	2017/5/10	
*Mauritia eglantina* (Duclos, 1833)	Cypraeidae	112	Hon La	108.44516	15.96868	2017/5/10	+
*Monetaria annulus* (Linnaeus, 1758)	Cypraeidae	113	Hon Dai	108.47814	15.94558	2017/5/10	+
*Monetaria caputserpentis* (Linnaeus, 1758)	Cypraeidae	114	Hon Dai	108.47814	15.94558	2017/5/10	
*Monetaria moneta* (Linnaeus, 1758)	Cypraeidae	115	Hon Tai	108.53193	15.90513	2017/5/10	+
*Naria helvola* (Linnaeus, 1758)	Cypraeidae	116	Hon Dai	108.47814	15.94558	2017/5/10	
*Sabia conica* (Schumacher, 1817)	Hipponicidae	117	Hon La	108.44516	15.96868	2017/5/10	+
*Echinolittorina cf. tricincta* Reid, 2007	Littorinidae	48	Bai Ong	108.50021	15.960193	2017/5/11	+
*Echinolittorina pascua* (Rosewater, 1970)	Littorinidae	49	Bai Bac	108.49052	15.96038	2017/5/12	+
*Littoraria undulata* (Gray, 1839)	Littorinidae	50	Bai Huong	108.52849	15.930946	2017/5/10	+
*Mammilla mammata* (Röding, 1798)	Naticidae	118	Hon La	108.44516	15.96868	2017/5/10	+
*Distorsio reticularis* (Linnaeus, 1758)	Personidae	119	Hon Dai	108.47814	15.94558	2017/5/10	+
*Lotoria lotoria* (Linnaeus, 1758)	Ranellidae	120	Hon Tai	108.53193	15.90513	2017/5/10	+
*Euprotomus aratrum* (Röding, 1798)	Strombidae	122	Hon Dai	108.47814	15.94558	2017/5/10	+
*Lambis lambis* (Linnaeus, 1758)	Strombidae	121	Hon Dai	108.47814	15.94558	2017/5/10	
*Tonna galea* (Linnaeus, 1758)	Tonnidae	144	Hon Dai	108.47814	15.94558	2017/5/10	+
*Tonna lischkeana* (Küster, 1857)	Tonnidae	123	Hon La	108.44516	15.96868	2017/5/10	+
*Babylonia areolata* (Link, 1807)	Babyloniidae	56	Hon Dai	108.47814	15.94558	2017/5/10	+
*Euplica scripta* (Lamarck, 1822)	Columbellidae	57	Bai Bac	108.49052	15.96038	2017/5/12	+
*Pyrene punctata* (Bruguière, 1789)	Columbellidae	58	Bai Huong	108.52849	15.930946	2017/5/10	+
*Conus betulinus* Linnaeus, 1758	Conidae	140	Hon Dai	108.47814	15.94558	2017/5/10	+
*Conus cinereus* Hwass in Bruguière, 1792	Conidae	61	Hon Dai	108.47814	15.94558	2017/5/10	+
*Conus coronatus* Gmelin, 1791	Conidae	62	Hon Dai	108.47814	15.94558	2017/5/10	+
*Conus ebraeus* Linnaeus, 1758	Conidae	63	Hon Tai	108.53193	15.90513	2017/5/10	+
*Conus flavidus* Lamarck, 1810	Conidae	64	Hon Dai	108.47814	15.94558	2017/5/10	+
*Conus lividus* Hwass in Bruguière, 1792	Conidae	65	Hon Tai	108.53193	15.90513	2017/5/10	
*Conus miles* Linnaeus, 1758	Conidae	66	Hon Tai	108.53193	15.90513	2017/5/10	+
*Conus nanus* G. B. Sowerby I, 1833	Conidae	67	Hon Dai	108.47814	15.94558	2017/5/10	+
*Conus quercinus* Lightfoot, 1786	Conidae	68	Hon Dai	108.47814	15.94558	2017/5/10	+
*Conus* sp.	Conidae	69	Hon Tai	108.53193	15.90513	2017/5/10	+
*Vexillum taeniatum* (Lamarck, 1811)	Costellariidae	60	Hon Tai	108.53193	15.90513	2017/5/10	+
*Vexillum* sp.	Costellariidae	59	Bai Huong	108.52849	15.930946	2017/5/10	+
*Latirus polygonus* (Gmelin, 1791)	Fasciolariidae	70	Hon Dai	108.47814	15.94558	2017/5/10	
*Peristernia nassatula* (Lamarck, 1822)	Fasciolariidae	71	Hon Dai	108.47814	15.94558	2017/5/10	+
*Pleuroploca trapezium* (Linnaeus, 1758)	Fasciolariidae	72	Hon La	108.44516	15.96868	2017/5/10	+
*Strigatella scutulata* (Gmelin, 1791)	Mitridae	73	Hon Tai	108.53193	15.90513	2017/5/10	+
*Chicoreus brunneus* (Link, 1807)	Muricidae	74	Bai Bac	108.49052	15.96038	2017/5/12	
*Chicoreus microphyllus* (Lamarck, 1816)	Muricidae	75	Bai Ong	108.50021	15.960193	2017/5/11	+
*Coralliophila erosa* (Röding, 1798)	Muricidae	141	Bai Ong	108.50021	15.960193	2017/5/11	+
*Coralliophila violacea* (Kiener, 1836)	Muricidae	76	Hon Tai	108.53193	15.90513	2017/5/10	+
*Drupa morum* Röding, 1798	Muricidae	77	Bai Ong	108.50021	15.960193	2017/5/11	+
*Drupa ricinus* (Linnaeus, 1758)	Muricidae	78	Bai Huong	108.52849	15.930946	2017/5/10	
*Drupa* sp.	Muricidae	79	Bai Huong	108.52849	15.930946	2017/5/10	+
*Drupella margariticola* (Broderip in Broderip & Sowerby, 1833)	Muricidae	80	Bai Bac	108.49052	15.96038	2017/5/12	+
*Mancinella alouina* (Röding, 1798)	Muricidae	81	Hon Dai	108.47814	15.94558	2017/5/10	
*Mancinella echinulata* (Lamarck, 1822)	Muricidae	82	Hon Tai	108.53193	15.90513	2017/5/10	+
*Murex trapa* Röding, 1798	Muricidae	83	Hon Dai	108.47814	15.94558	2017/5/10	+
*Nassa francolina* (Bruguière, 1789)	Muricidae	84	Hon La	108.44516	15.96868	2017/5/10	+
*Oppomorus purpureocinctus* (Preston, 1909	Muricidae	85	Bai Ong	108.50021	15.960193	2017/5/11	+
*Oppomorus funiculatus* (Reeve, 1846)	Muricidae	86	Bai Bac	108.49052	15.96038	2017/5/12	+
*Purpura panama* (Röding, 1798)	Muricidae	87	Hon Tai	108.53193	15.90513	2017/5/10	+
*Rapana rapiformis* (Born, 1778)	Muricidae	88	Hon Dai	108.47814	15.94558	2017/5/10	+
*Reishia cf. bronni* (Dunker, 1860)	Muricidae	89	Hon La	108.44516	15.96868	2017/5/10	+
*Reishia cf. clavigera* (Küster, 1860)	Muricidae	90	Hon Dai	108.47814	15.94558	2017/5/10	+
*Tenguella granulata* (Duclos, 1832)	Muricidae	91	Bai Ong	108.50021	15.960193	2017/5/11	+
*Tenguella musiva* (Kiener, 1835)	Muricidae	92	Bai Bac	108.49052	15.96038	2017/5/12	+
*Thais* sp.	Muricidae	93	Bai Huong	108.52849	15.930946	2017/5/10	+
*Tylothais virgata* (Dillwyn, 1817)	Muricidae	94	Hon Dai	108.47814	15.94558	2017/5/10	+
*Pollia undosa* (Linnaeus, 1758)	Pisaniidae	124	Hon Dai	108.47814	15.94558	2017/5/10	
*Vasum turbinellus* (Linnaeus, 1758	Turbinellidae	95	Hon Tai	108.53193	15.90513	2017/5/10	
*Gemmula* sp.	Turridae	96	Hon Tai	108.53193	15.90513	2017/5/10	+
*Melo melo* (Lightfoot, 1786)	Volutidae	97	Hon La	108.44516	15.96868	2017/5/10	+
*Herpetopoma instrictum* (Gould, 1849)	Chilodontaidae	125	Hon La	108.44516	15.96868	2017/5/10	+
*Siphonaria* sp.	Siphonariidae	126	Hon Tai	108.53193	15.90513	2017/5/10	+
*Tectus pyramis* (Born, 1778)	Tegulidae	127	Hon Tai	108.53193	15.90513	2017/5/10	
*Tegula* sp.	Tegulidae	128	Bai Huong	108.52849	15.930946	2017/5/10	+
*Monodonta labio* (Linnaeus, 1758)	Trochidae	129	Bai Bac	108.49052	15.96038	2017/5/12	+
*Trochus maculatus* Linnaeus, 1758	Trochidae	130	Hon Dai	108.47814	15.94558	2017/5/10	
*Trochus stellatus* Gmelin, 1791	Trochidae	131	Hon Tai	108.53193	15.90513	2017/5/10	+
*Umbonium vestiarium* (Linnaeus, 1758)	Trochidae	132	Hon La	108.44516	15.96868	2017/5/10	+
*Turbo chrysostomus* Linnaeus, 1758	Turbinidae	133	Hon Dai	108.47814	15.94558	2017/5/10	
*Turbo bruneus* (Röding, 1798)	Turbinidae	134	Hon Dai	108.47814	15.94558	2017/5/10	+
*Cerithium punctatum* Bruguière, 1792	Cerithiidae	99	Bai Bac	108.49052	15.96038	2017/5/12	+
*Cerithium traillii* G. B. Sowerby II, 1855	Cerithiidae	100	Bai Ong	108.50021	15.960193	2017/5/11	+
*Clypeomorus bifasciata* (G. B. Sowerby II, 1855)	Cerithiidae	101	Hon La	108.44516	15.96868	2017/5/10	+
*Clypeomorus petrosa* chemnitziana (Pilsbry, 1901)	Cerithiidae	102	Bai Ong	108.50021	15.960193	2017/5/11	+
*Rhinoclavis sinensis* (Gmelin, 1791)	Cerithiidae	103	Hon La	108.44516	15.96868	2017/5/10	+
*Lottia* sp.	Lottiidae	135	Hon Tai	108.53193	15.90513	2017/5/10	+
*Patelloida cf. saccharina* (Linnaeus, 1758)	Lottiidae	136	Hon Tai	108.53193	15.90513	2017/5/10	+
*Cellana* sp.	Nacellidae	137	Bai Huong	108.52849	15.930946	2017/5/10	
*Scutellastra flexuosa* (Quoy & Gaimard, 1834)	Patellidae	138	Hon La	108.44516	15.96868	2017/5/10	+
*Terebralia sulcata* (Born, 1778)	Potamididae	53	Bai Huong	108.52849	15.930946	2017/5/10	+
