# Supplementary material for: Sample checklist of Gastropoda and Bivalvia in Cham Islands, Vietnam
Source: Biodivers Data J. 2019 Feb 19;(7):e32930. doi: 10.3897/BDJ.7.e32930 (PMC6391368; doi:10.3897/BDJ.7.e32930)
Supplement: Supplementary material 2 — Some photos of the species collected from Cham Islands, Vietnam, in May 2017 [file bdj-07-e32930-s002.pdf]

Some photos of the species collected from Cham Islands, Vietnam, in May 2017

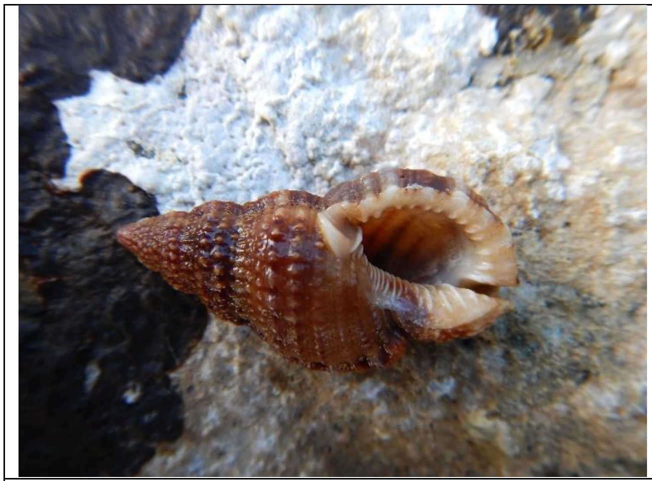	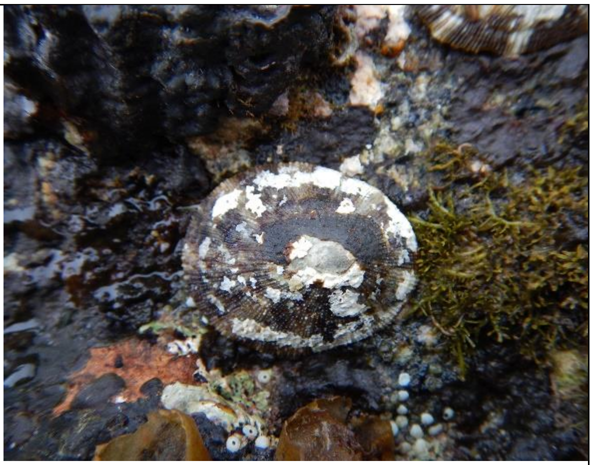
Bursa granularis	*Cellana* sp.
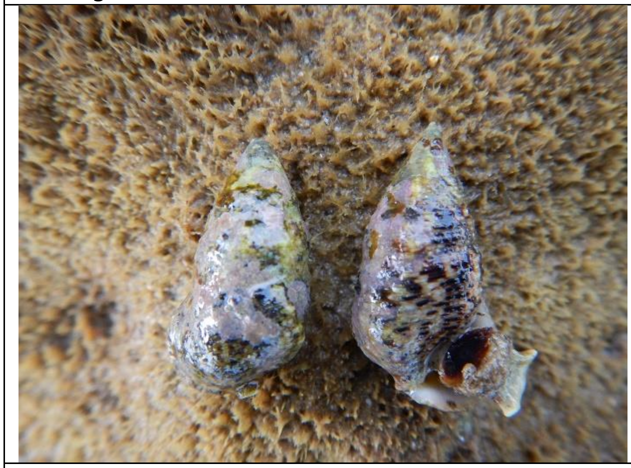	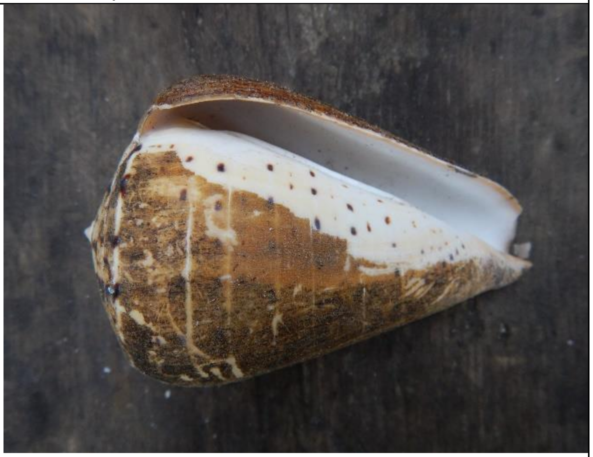
Clypeomorus petrosa chemnitziana	*Conus betulinus*
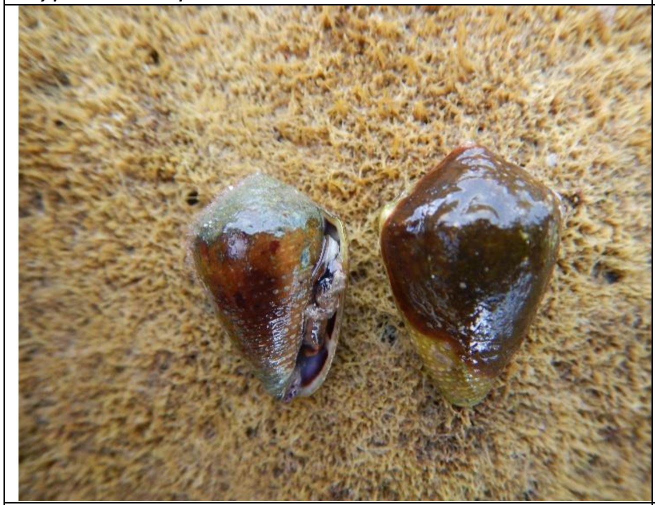	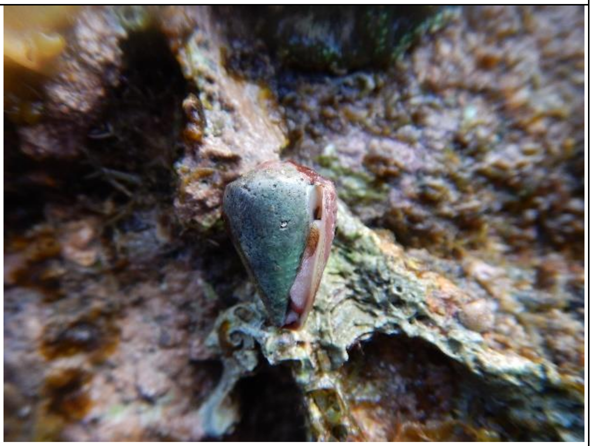
Conus coronatus	*Conus nanus*

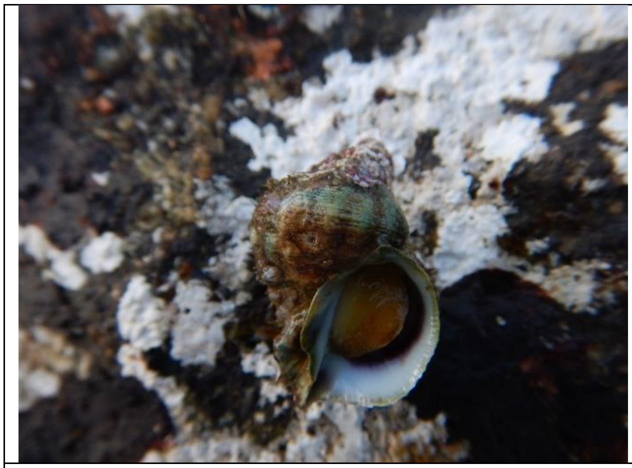

Coralliophila erosa

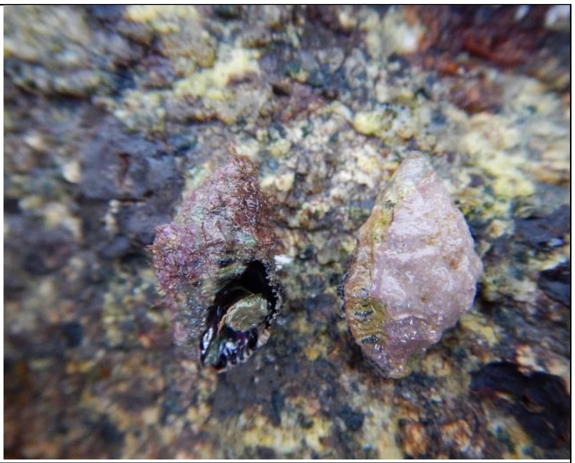

Drupella margariticola

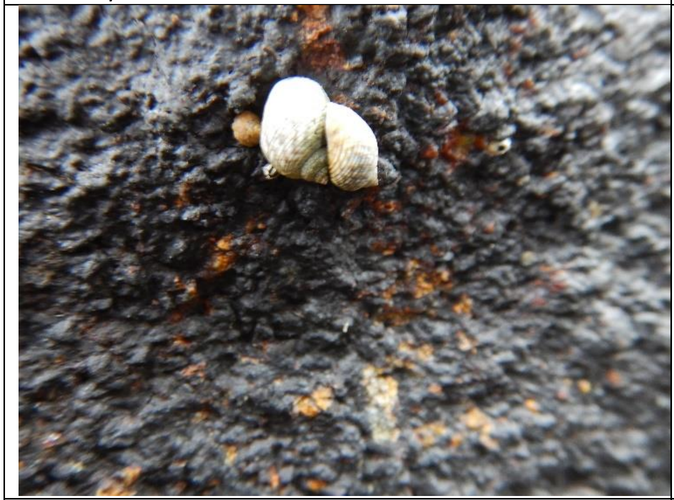

Echinolittorina cf. tricincta

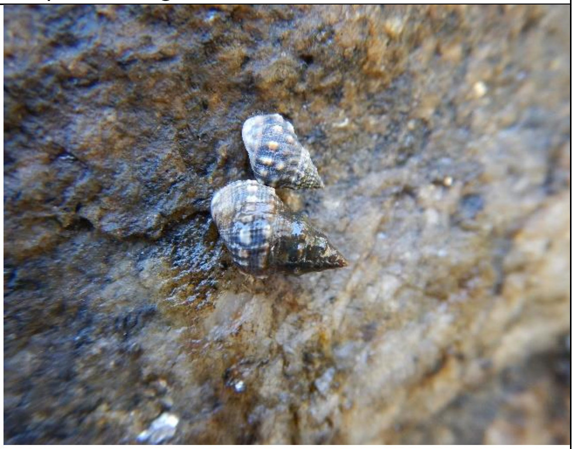

Echinolittorina pascua

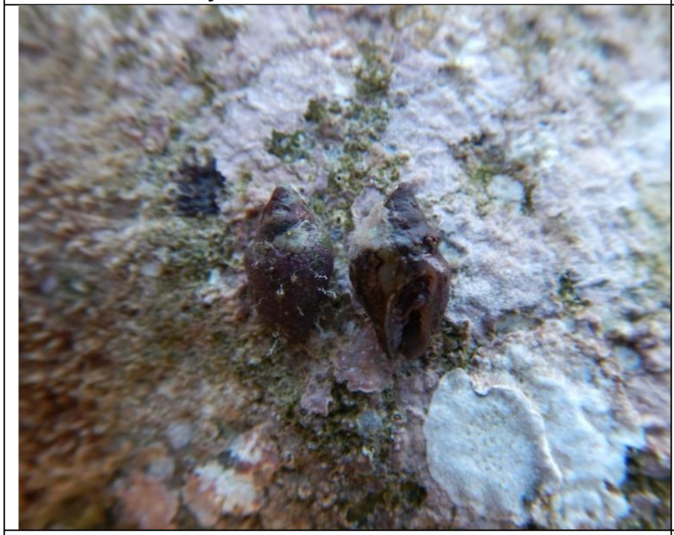

Euplica scripta

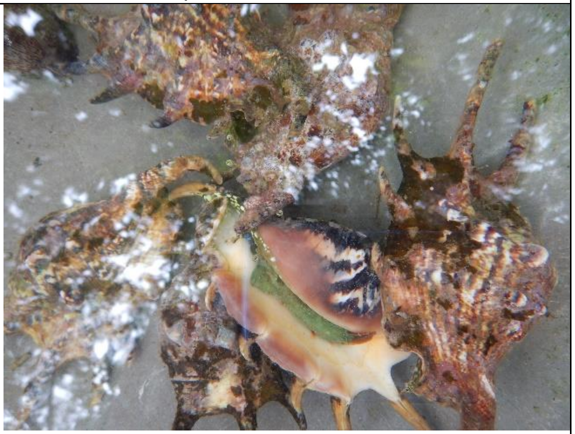

Lambis lambis

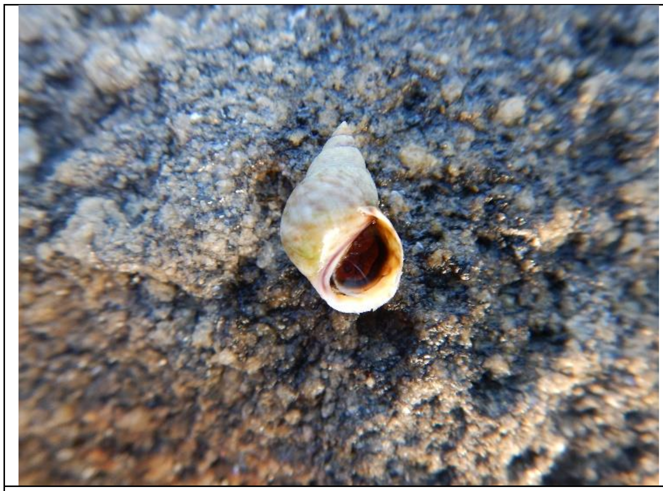

Littoraria undulata

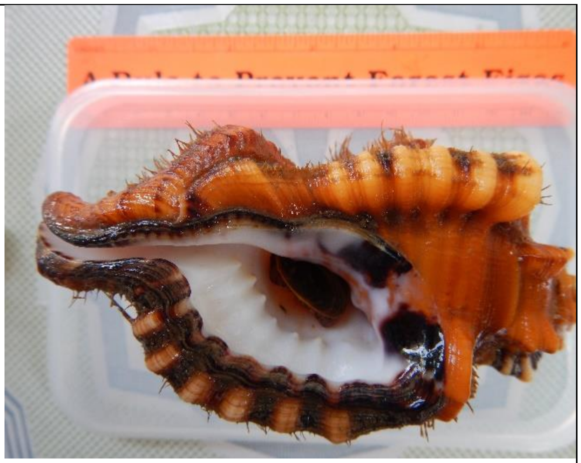

Lotoria lotoria

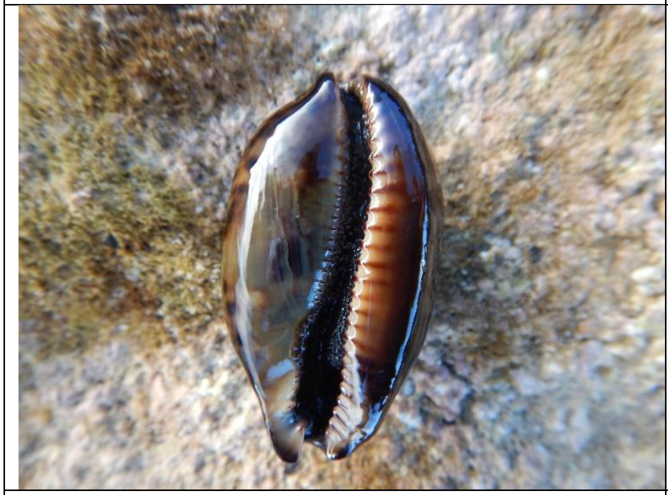

Mauritia eglantina

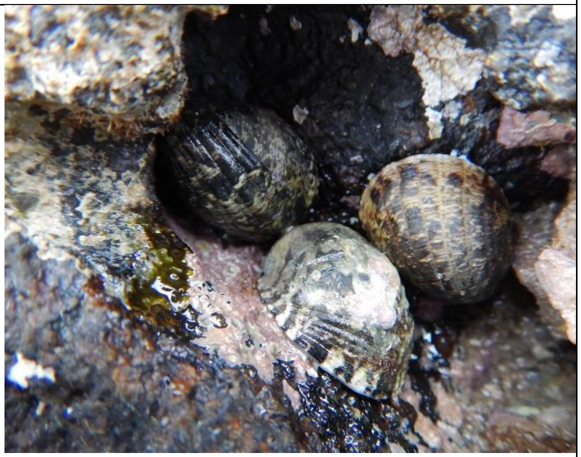

Nerita albicilla

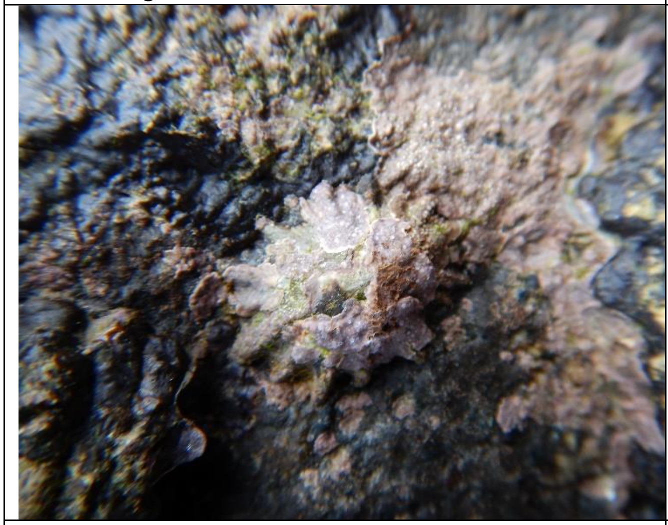

Patelloida cf. saccharina

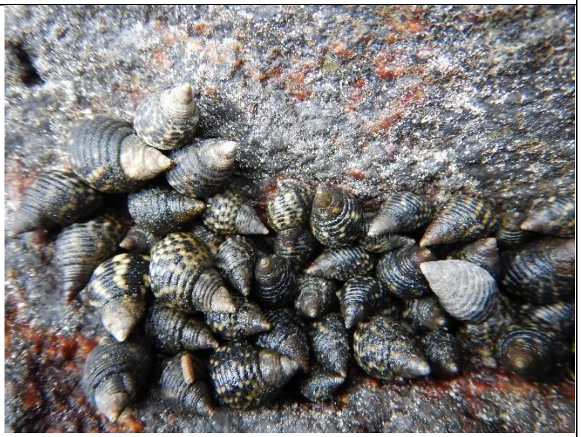

Planaxis sulcatus

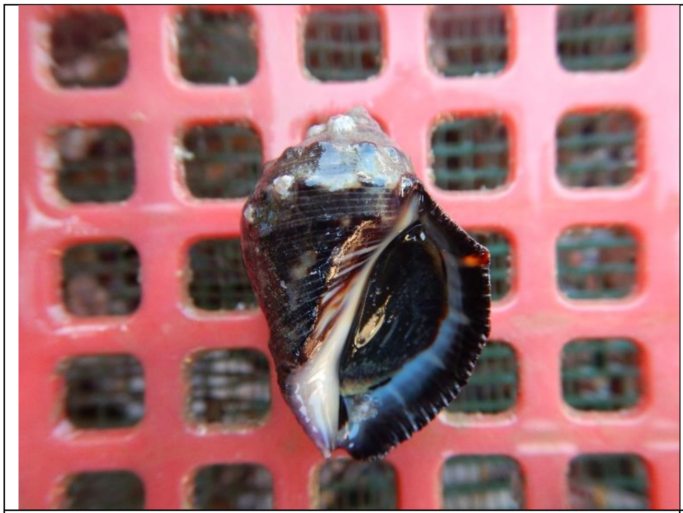	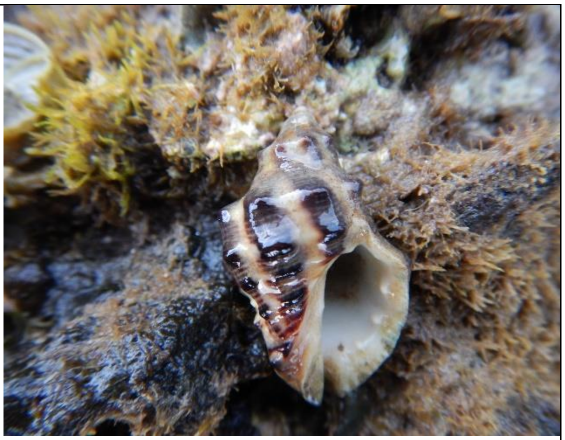
Purpura panama	*Reishia cf. bronni*
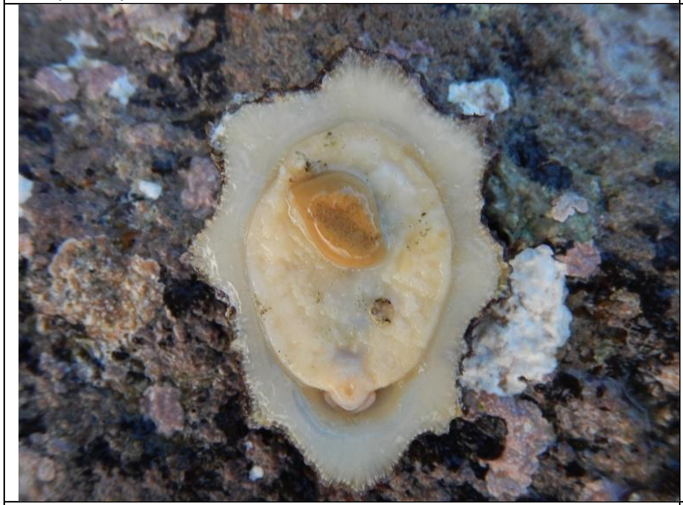	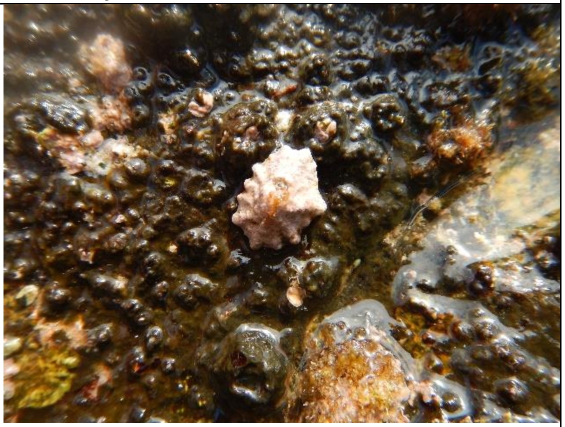
Scutellastra flexuosa	*Siphonaria* sp.
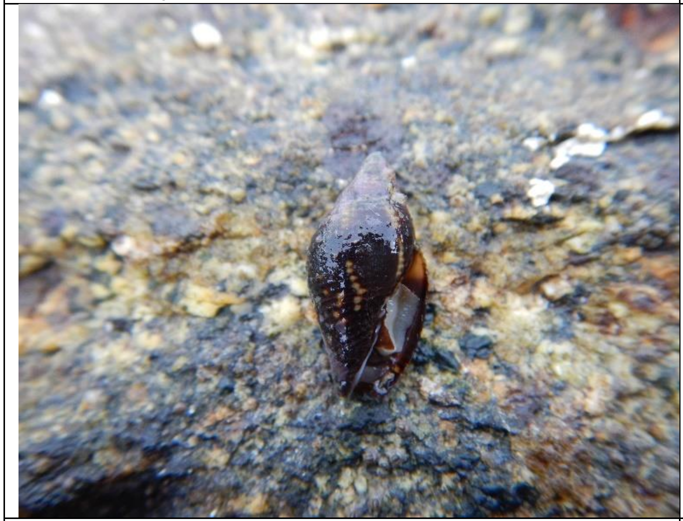	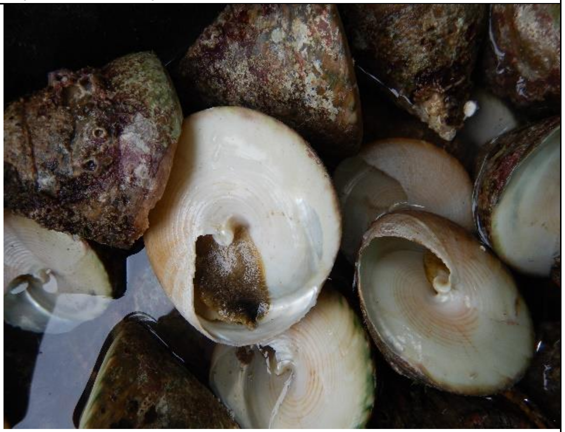
Strigatella scutulata	*Tectus pyramis*

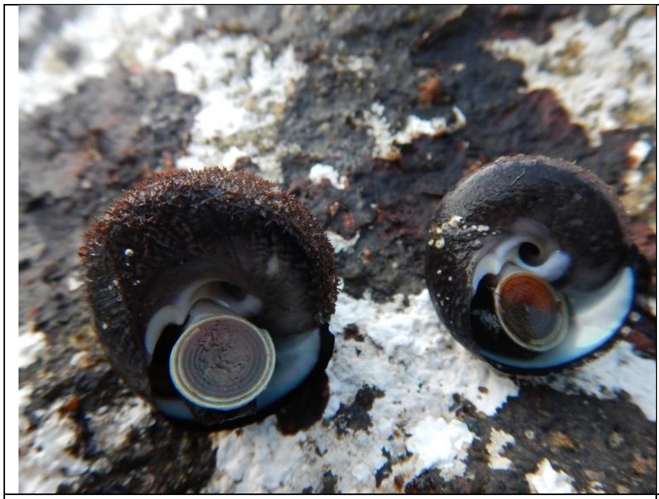	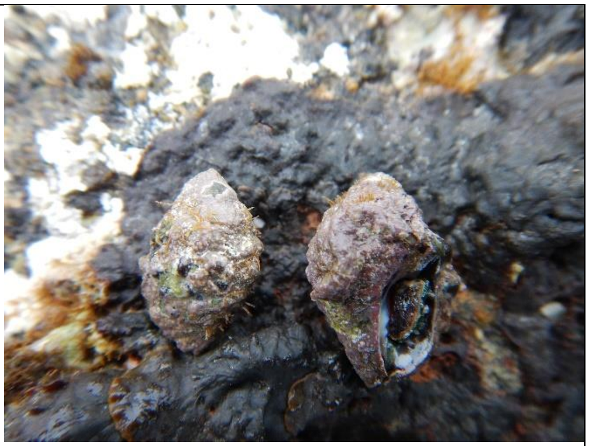
Tegula sp.	*Tenguella granulata*
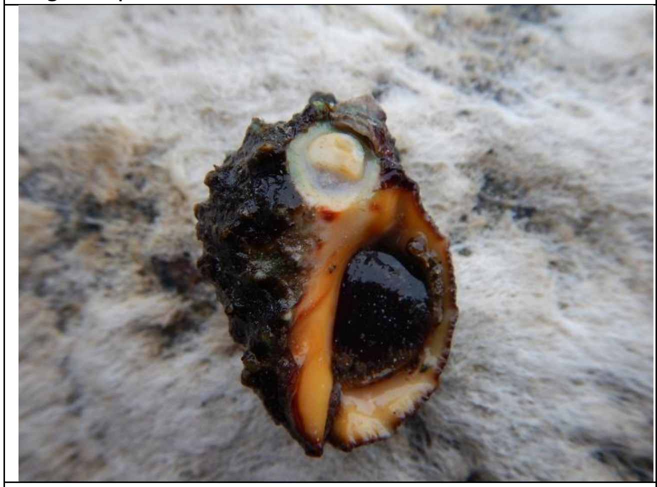	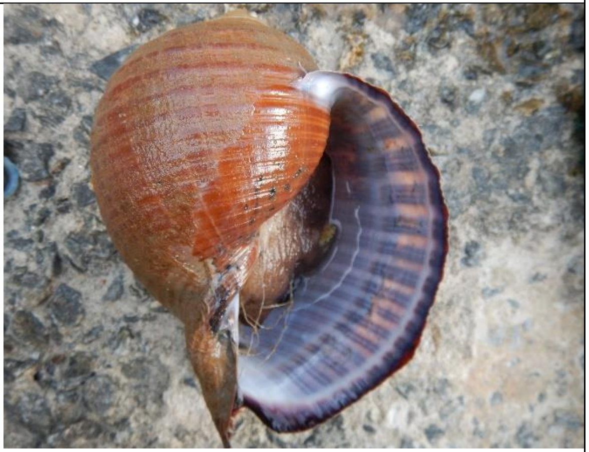
Thais sp.	*Tonna galea*
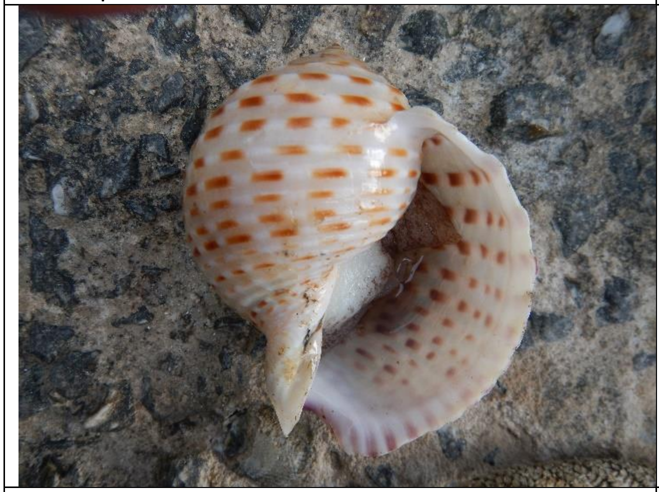	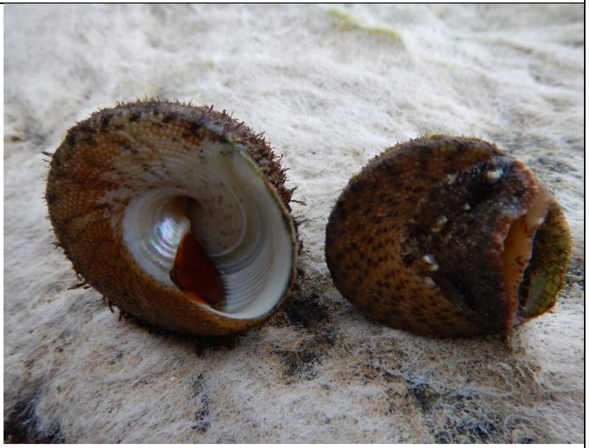
Tonna lischkeana	*Trochus maculatus*

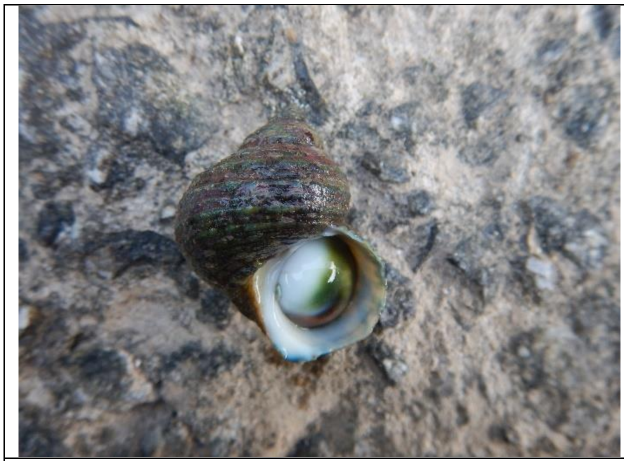

Turbo bruneus

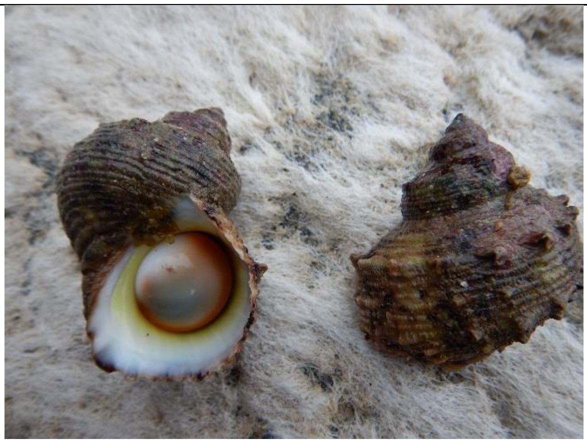

Turbo chrysostomus

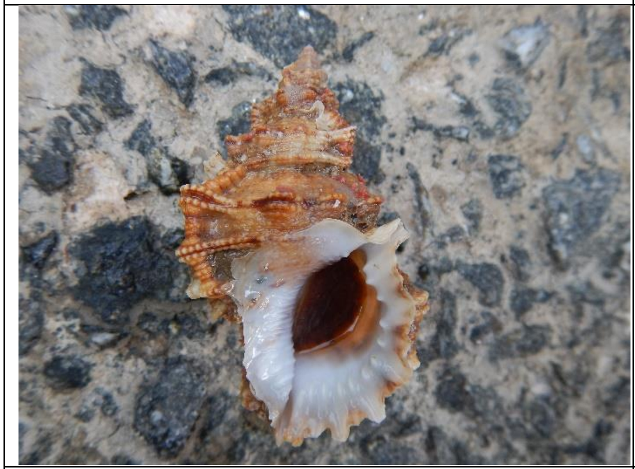

Tutufa oyamai

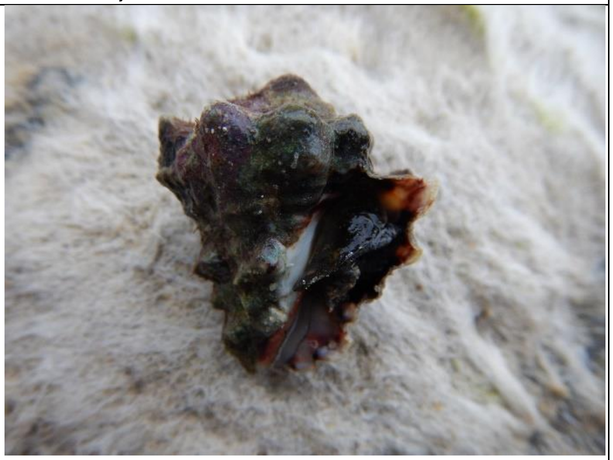

Tylothais virgata

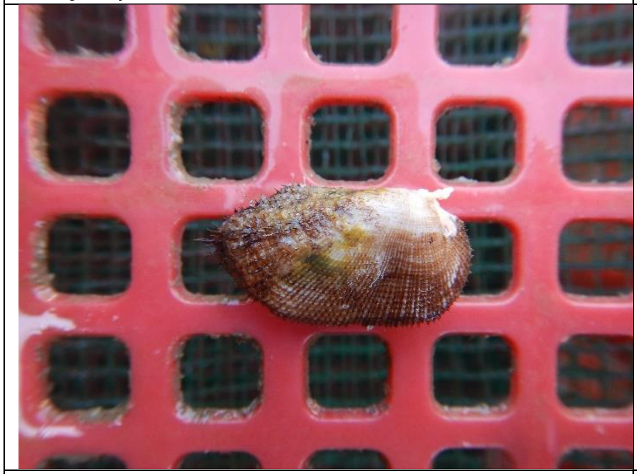

Barbatia foliata

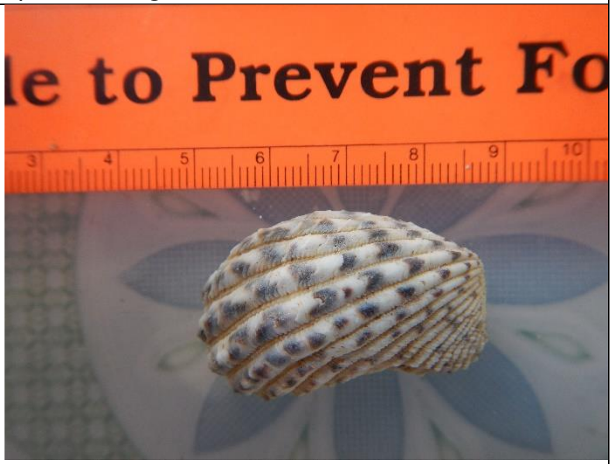

Cardita variegata

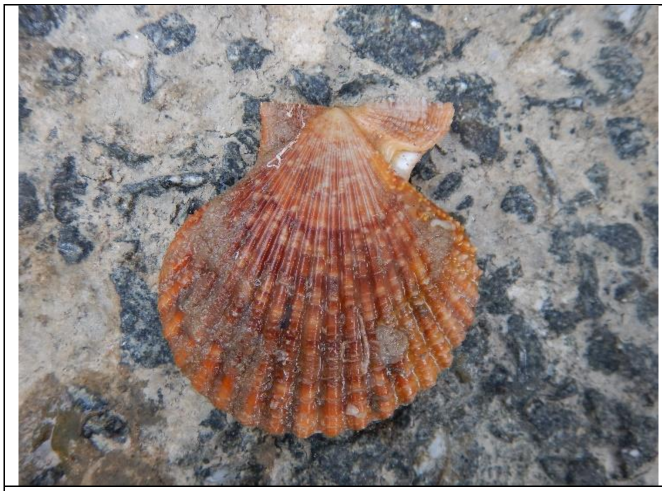

Mimachlamys sanguinea

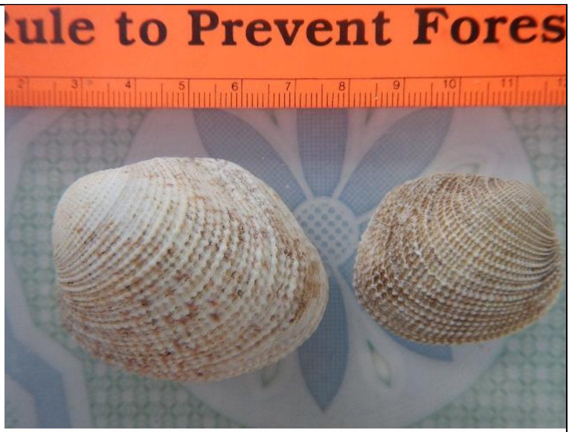

Periglypta albocancellata

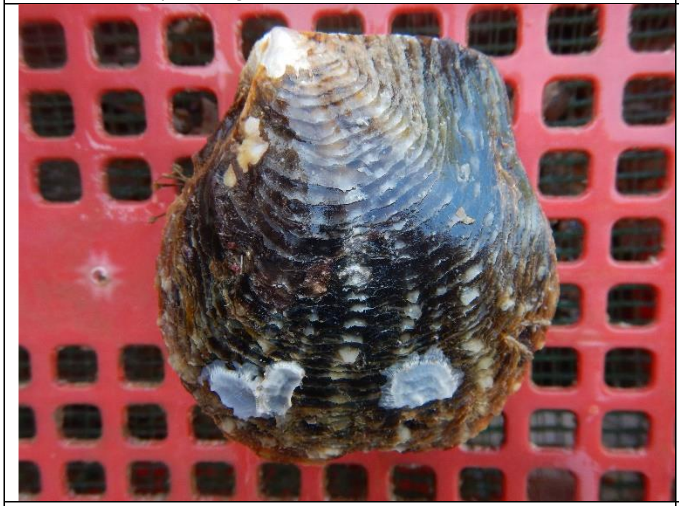

Pinctada margaritifera

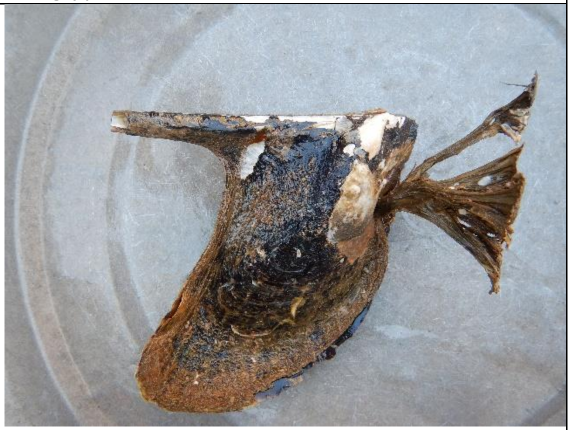

Pteria penguin
